# A Multi-State, Cross-Sectional Retrospective Study on Knowledge, Attitude and Practice of Rabies Post-Exposure Prophylaxis Including Rabies Monoclonal Antibody Among Physicians Across India

**DOI:** 10.3390/vaccines14070635

**Published:** 2026-07-20

**Authors:** Ravish Haradanahalli Shankaraiah, Navyashree N. S., Divya Bharathi G., Trayambak Dutta, Manish Mahajan

**Affiliations:** 1 Department of Community Medicine, Kempegowda Institute of Medical Sciences, Bangalore 560070, India; drravishhs@rediffmail.com (R.H.S.); dr.navyashreens@gmail.com (N.N.S.); drdivyabharathig@gmail.com (D.B.G.); 2 Vaccines, Zydus Life Sciences Ltd., Ahmedabad 382481, India; 3 Biologics & Vaccines, Zydus Lifesciences Ltd., Ahmedabad 382481, India; manish.mahajan@zyduslife.com

**Keywords:** rabies, post-exposure prophylaxis, KAP, rabies monoclonal antibodies, physicians

## Abstract

Background/Objectives: Rabies remains a lethal vaccine-preventable disease in India, and physicians managing animal-bite cases are central to effective post-exposure prophylaxis (PEP). With the increasing use of rabies monoclonal antibodies (RmAbs) as alternatives to conventional rabies immunoglobulins, this study assessed physicians’ knowledge, attitude, and practice (KAP) regarding rabies PEP, including RmAb awareness and acceptance. Methods: This multi-state, cross-sectional retrospective KAP survey was conducted among 208 physicians across ten Indian states using a structured, self-administered Google Form. The 33-item questionnaire assessed Knowledge, Attitude, and Practice domains, with 11 items each, scored binarily according to NCDC guidelines. Descriptive statistics, Shapiro–Wilk normality testing, Kruskal–Wallis tests with post hoc corrections, and Spearman rank-order correlations were performed. Results: Mean domain scores were 8.00/11 for Knowledge (72.7%), 7.22/11 for Attitude (65.6%), and 8.64/11 for Practice (78.5%). Key gaps were identified in the intramuscular pre-exposure prophylaxis schedule, management of excess RIG/RmAb after wound infiltration, exposure categorization of animal contact, and vaccine administration site in young children. The Knowledge–Practice correlation was moderately strong and statistically significant (r = 0.520, *p* < 0.001). Acceptance of RmAbs was high, with 88.5% recognizing them as effective replacements for RIGs and 84.6% considering them safer than RIGs. Practice scores showed the most consistent improvement, Knowledge scores improved on most items but declined sharply for the pre-exposure prophylaxis schedule, and Attitude scores showed a mixed pattern of gains and losses compared with the 2013 benchmark survey. Conclusions: Overall KAP scores were adequate, with Practice scoring highest. Targeted continuing medical education focused on NCDC guidelines and practical PEP scenarios is needed among physicians managing rabies exposures.

## 1. Introduction

Rabies is a fatal viral zoonosis caused by neurotropic lyssaviruses. The disease affects approximately 59,000 human lives annually and 96% of deaths occur in Africa and Asia despite the availability of highly effective vaccines and passive immunization agents [1]. India reports an estimated 20,565 deaths per year and ~17.4 million animal bites annually. Of these, domestic dogs account for more than 96.2% of human rabies exposures in urban settings [2].

Category-wise data from multicentric Indian surveillance indicate that Category III exposures, which require the complete PEP triad including passive immunization, account for over half of all reported animal-bite presentations, Category II exposures for most of the remainder, and Category I (non-exposures not requiring PEP) for only a small minority [3]. This distribution means that a substantial proportion of physician-patient encounters in Indian anti-rabies clinics require accurate exposure categorization and correct selection of a passive immunizing agent, RIG or RmAb, underscoring why physician-level KAP gaps in exposure categorization and RIG/RmAb use carry direct clinical consequences at population scale.

Rabies-associated deaths can be prevented by timely and appropriate administration of post-exposure prophylaxis (PEP). World Health Organization (WHO) recommended a complete PEP regimen for Category III exposures, which comprises thorough wound washing with soap and water, a full course of cell-culture anti-rabies vaccine (ARV), and passive immunization with rabies immunoglobulin (RIG) or the newer rabies monoclonal antibodies (RmAbs) [4]. Adherence to PEP by frontline physicians at the anti-rabies clinic is therefore the single most critical determinant for the prevention of rabies-related fatalities.

A benchmark Indian study by Shankaraiah et al. (2013) assessed the knowledge, attitude, and practice of 109 physicians at animal bite clinics in eight Indian cities and identified substantial gaps in all three domains, which include inadequate wound categorization, inappropriate use of RIG, and poor compliance with intradermal vaccination schedules [5]. The study concluded that there is an urgent need for repeated CMEs of physicians aligned with WHO guidelines.

A significant development in India is the regulatory approval of rabies monoclonal antibody preparations, i.e., Rabishield (2016) and TwinRab (2019), as alternatives to conventional RIGs for passive immunization in Category III exposures. RmAbs have advantages such as standardized recombinant production with high batch-to-batch consistency, reduced risk of serum sickness and hypersensitivity reactions associated with animal or plasma-derived immunoglobulins, elimination of the risk of blood-borne pathogen contamination, and smaller infiltration volumes due to higher specific potency compared to conventional RIGs [6].

The WHO has formally endorsed RmAbs as an alternative to RIG for passive immunization in Category III exposures. However, the National Centre for Disease Control (NCDC) guidelines for Rabies Prophylaxis (2019) did not incorporate this recommendation, which is a recognized gap between global guidance and Indian national policy [7]. Whether this paradigm shift is reflected in physician knowledge and practice has not been assessed nationally.

KAP surveys are a widely used epidemiological tool in public health research for assessing the gap between theoretical knowledge, attitudinal beliefs, and actual clinical practice, and for guiding the design of targeted health education interventions [8]. Existing Indian KAP literature on rabies prophylaxis has largely been limited in geographic representation and sample size, and all identified studies predate the regulatory approval of RmAbs in India, leaving a gap in evidence regarding physician knowledge and practice in the current era of evolving passive immunization options [5]. A comprehensive, multi-state assessment incorporating RmAb awareness alongside core PEP knowledge, attitude, and practice domains is therefore warranted.

Rabies KAP among physicians also has direct relevance to India’s One Health approach. India’s National Action Plan for Dog-Mediated Rabies Elimination (NAPRE) by 2030 integrates human, animal, and environmental health sectors, recognizing that sustainable rabies control depends on coordinated action across mass dog vaccination, dog population management, and timely human PEP [9]. Within this framework, physician KAP regarding PEP represents the human-health pillar most directly linked to preventing rabies deaths once an exposure has already occurred, complementing veterinary and environmental measures aimed at reducing exposure incidence. Strengthening physician-level knowledge, attitude, and practice is therefore an integral part of, rather than separate from, India’s One Health rabies elimination effort.

Physician KAP can plausibly influence the administration of RmAbs in routine PEP delivery in several ways. Accurate knowledge of RmAb dosing and indications is a prerequisite for prescribing them in place of conventional RIGs; favourable attitudes toward RmAbs as safe and effective alternatives may reduce clinician hesitancy in settings where both agents are available; and once established, consistent practice patterns are more likely to persist as RmAbs are progressively incorporated into institutional and national protocols. Establishing the current KAP baseline for RmAbs is therefore a necessary step toward understanding and ultimately increasing their administration among physicians managing rabies exposures across India.

The objectives of this study were: (i) to assess current Knowledge, Attitude, and Practice of Indian physicians regarding rabies PEP, including RmAb awareness; (ii) to examine inter-domain correlations between Knowledge, Attitude, and Practice; and (iii) to identify speciality- and state-level differences to guide targeted educational interventions.

## 2. Materials and Methods

### 2.1. Study Design and Setting

This multi-state, cross-sectional retrospective KAP survey was conducted using a structured, self-administered questionnaire distributed electronically via Google Forms to physicians involved in rabies post-exposure prophylaxis across multiple Indian states. Recruitment was conducted through the APCRI (Association for Prevention and Control of Rabies in India) conference platform. All physicians registered on this platform were invited to participate; participation was entirely voluntary, and physicians who provided informed consent electronically prior to completing the questionnaire were included in the analytic cohort. The study team did not select participants individually and had no control over the number of respondents contributed by any given state; the resulting state-wise distribution therefore reflects self-selected registration and consent rather than a stratified or quota-based sampling design. Because recruitment depended on voluntary conference registration and consent rather than a defined sampling frame of practicing physicians per state, a state-level response rate could not be calculated and proportional representation across states could not be guaranteed; states with larger anti-rabies clinic networks or higher conference attendance are likely over-represented relative to their physician population. An aggregate response rate for the full pool of APCRI-registered physicians could similarly not be calculated, for the same reason. To reduce the likelihood of duplicate submissions, the Google Form was configured to accept only one submission per respondent and participants were asked to confirm that they had not previously completed the survey; because responses were not linked to a verified unique identifier such as a medical registration number; however, the possibility of a small number of undetected duplicate entries cannot be entirely excluded.

### 2.2. Study Population and Sampling

Eligible participants were physicians actively involved in animal-bite and rabies PEP management at the anti-rabies clinic, spanning community medicine, surgery, emergency medicine, pediatrics, general medicine and other related specialties. A target sample of approximately 200 participants was pre-specified based on feasibility. In the absence of a validated national prevalence estimate for KAP indicators among Indian physicians against which a formal power calculation could be anchored, the target sample size was set pragmatically at approximately double the 2013 benchmark cohort of 109 physicians [5], with the aim of improving precision around domain-level estimates, enabling meaningful speciality- and state-level stratification, and supporting adequately powered non-parametric between-group comparisons. This approach follows common practice in exploratory, multi-state KAP surveys where a pre-existing sampling frame of eligible physicians is not centrally available.

### 2.3. Questionnaire Structure

The 33-item questionnaire was structured across three domains, which were aligned as per NCDC 2019 and WHO 2018 guidelines [4,10]:

Knowledge (11 items; maximum score = 11) covered rabies-transmitting animals, WHO/NCDC exposure categorization, ARV dosing (infants, pregnancy), IM PEP and pre-exposure prophylaxis (PrEP) schedules, RIG, single and cocktail RmAb dosing, and management of excess RIG/ RmAb after wound infiltration.

Attitude (11 items; maximum score = 11) evaluated evidence-based beliefs on animal transmission, Category II exposure classification, wound management, vaccine route equivalence and acceptance of RmAbs as alternative passive-immunization agents.

Practice (11 items; maximum score = 11) assessed clinical decision-making via scenario-based items covering PEP in non-canine bites, pregnancy, pediatric patients, previously vaccinated individuals, delayed presentations, post-vaccination serology referral, and Category III management algorithms.

### 2.4. Questionnaire Validation

The questionnaire’s item structure was aligned with NCDC 2019 and WHO 2018 guidelines, and its item content was matched, where possible, to the domains and scoring approach used in the 2013 benchmark study by Shankaraiah et al. [5] to support comparability over time. A formal pilot test of the instrument and a quantitative assessment of internal consistency (e.g., Cronbach’s alpha) were not performed prior to administration. This is acknowledged as a limitation of the present study; formal psychometric validation, including pilot testing and reliability assessment, is recommended before any future iteration of this instrument is deployed more widely.

### 2.5. Scoring

Each Knowledge and Practice item was scored 1 (correct) or 0 (incorrect or “Don’t know”) per the NCDC 2019 answer key [10]. Attitude items were scored 1 only if the response (Agree or Disagree) aligned with the evidence-based guideline stance; “Don’t know” responses were scored 0. Domain totals ranged 0 to 11; mean percentages were calculated as (mean score/11) × 100.

### 2.6. Statistical Analysis

Descriptive statistics (mean ± standard deviation [SD]) were computed for each domain. The Shapiro–Wilk test was applied to assess normality; all three distributions significantly deviated from normality due to the scoring system (*p* < 0.0001), justifying non-parametric analysis throughout. Kruskal–Wallis tests assessed speciality and state-level differences; while significant findings were followed by Mann–Whitney U post hoc tests with Bonferroni correction. Spearman rank-order correlations (r) were computed for K-A, K-P, and A-P domain pairs, both overall and stratified by speciality and state. The 2013 study by Shankaraiah et al. [5] was selected as the historical benchmark because it used a comparable item-level questionnaire structure covering the same three KAP domains among Indian physicians managing animal-bite exposures, providing the closest available matched-item comparator for assessing temporal change; no other Indian physician-level KAP dataset with matched items and comparable scoring was identified. For comparative analysis with the 2013 benchmark study, two-proportion Z-tests were used for item-level comparisons, Mann–Whitney U tests were used for domain-level score comparisons, and the Fisher r-to-z transformation was applied to compare Spearman correlation coefficients. A significance threshold of *p* < 0.05 was applied throughout. Analyses were performed in SPSS v25.0.

Because the 2013 and 2026 cohorts were independently sampled non-probability convenience samples rather than a matched longitudinal cohort, this comparison is susceptible to several forms of bias. Selection bias may arise because the two studies drew on different, non-random pools of physicians (eight cities in 2013 versus ten states with broader specialty representation in 2026), so observed differences may partly reflect differences in who chose to participate rather than true secular change. Information bias may arise from the shift in survey mode (interviewer- or paper-based administration in 2013 versus a self-administered Google Form in 2026), which can affect social-desirability and recall patterns differently across the two samples. Outcome-assessment bias is minimized to the extent that matched items were scored against the same NCDC/WHO answer key in both studies, but residual differences in question wording or context cannot be fully excluded. These comparisons should therefore be interpreted as indicative of broad temporal trends. Because the 2013 and 2026 studies are two independent cross-sectional samples rather than a longitudinal cohort of the same physicians, observed differences between them are associations over calendar time, not a measured, causally attributable improvement or decline; such differences are therefore described as being “observed” or “differing from” the 2013 benchmark rather than as physicians’ knowledge or practice having directly “improved” or “worsened” as a result of any specific intervention.

### 2.7. Ethical Considerations

The study was approved by the ACEAS-Independent Ethics Committee, Ahmedabad, Gujarat, India, on 15 January 2026 (Protocol No. PNRAB052026024). The study was conducted in accordance with the Declaration of Helsinki, Good Clinical Practice guidelines, Indian Council of Medical Research (ICMR) guidelines, and New Drugs Clinical Trials Rules 2019 and its 2023 amendment. Written informed consent was obtained from participants prior to study participation, where applicable.

## 3. Results

### 3.1. Participant Characteristics

A total of 208 physicians completed the survey (Table 1). The largest speciality group was Community Medicine (*n* = 71, 34.1%), followed by Emergency Medicine (*n* = 58, 27.9%), Pediatricians (*n* = 44, 21.2%), General Physicians (*n* = 14, 6.7%), and other specialities including surgeons (*n* = 21, 10.1%). Karnataka contributed the most respondents (*n* = 45, 21.6%), followed by Uttar Pradesh (*n* = 37, 17.8%), Kerala (*n* = 22, 10.6%), Tamil Nadu (*n* = 15, 7.2%), and Chandigarh (*n* = 12, 5.8%). This specialty and state distribution characterizes the 2026 cohort and forms the basis for the specialty-composition comparison with the 2013 benchmark cohort.

### 3.2. Domain-Level Descriptive Statistics

All three domain distributions significantly deviated from normality (Shapiro–Wilk, all *p* < 0.0001), justifying non-parametric methods. Domain-level results are summarized in Table 2. Practice was the highest-scoring domain (78.5%), followed by Knowledge (72.7%) and Attitude (65.6%).

### 3.3. Knowledge Domain: Item-Level Analysis

**Figure 1:**
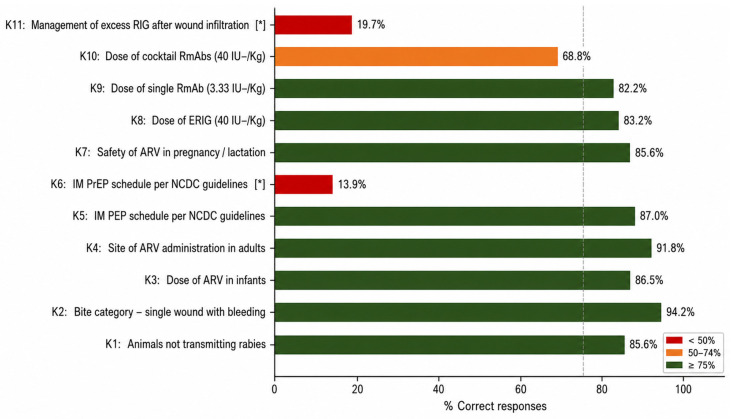
Knowledge Domain: Item-Level Correct Response Rates (*n* = 208). Green ≥75%, amber 50–74%, red <50%. Dashed Line Indicates the 75% Benchmark for Adequate Knowledge. * Critical Knowledge Gaps (Correct Response Rate < 50%).

Item-level correctness rates are presented in Table 3 and Figure 1. Strong knowledge was observed for exposure categorization (K2: 94.2%), ARV site in adults (K4: 91.8%), intramuscular (IM) PEP schedule (K5: 87.0%), and ARV safety in pregnancy (K7: 85.6%). Single-agent RmAb dosing was well known (K9: 82.2%), though cocktail dosing knowledge was lower (K10: 68.8%). Critical gaps were identified in the IM PrEP schedule (K6: 13.9%) and management of excess RIG after wound infiltration (K11: 19.7%).

### 3.4. Attitude Domain—Item-Level Analysis

**Figure 2:**
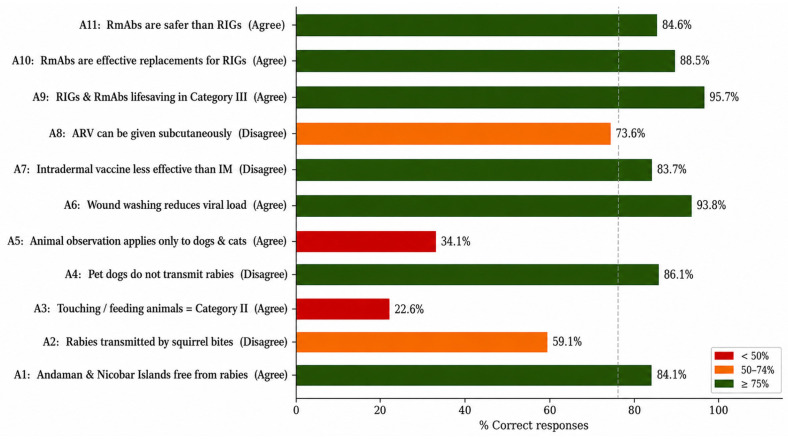
Attitude Domain: Item-Level Correct Response Rates (*n* = 208). Green ≥75%, amber 50–74%, red <50%. Dashed Line Indicates the 75% Benchmark for Adequate Attitude Scores.

Attitude item-level results are shown in Table 4 and Figure 2. Near-universal acceptance was recorded for wound washing (A6: 93.8%), RIGs/RmAbs as lifesaving agents (A9: 95.7%), and RmAbs as effective replacements for RIGs (A10: 88.5%) and safer alternatives (A11: 84.6%). Recognition that the intradermal vaccine is equivalent to the intramuscular (A7: 83.7%) was also strong. Major deficiencies were identified in classifying animal contact (touching or feeding) as Category I exposure (A3: 22.6%) and accepting the animal observation rule as applying only to dogs and cats (A5: 34.1%).

### 3.5. Practice Domain—Item-Level Analysis

**Figure 3:**
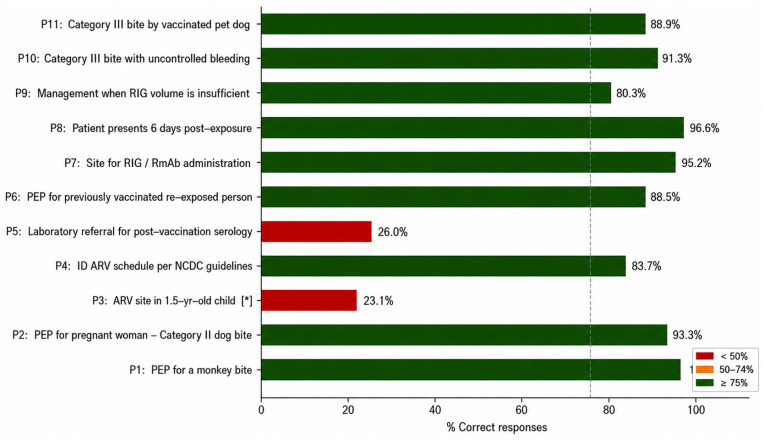
Practice Domain: Item-Level Correct Response Rates (*n* = 208). Green ≥75%, amber 50–74%, red <50%. Dashed Line Indicates the 75% Benchmark for Adequate Practice Scores. * Denotes Critical Practice Gap (Correct Response Rate < 50%).

Practice item-level results are presented in Table 5 and Figure 3. Near-universal correct practice was recorded for PEP following monkey bites (P1: 97.1%), management of delayed presentations (P8: 96.6%), RIG/ RmAb administration site (P7: 95.2%), and PEP in pregnant women with Category II bites (P2: 93.3%). Marked deficiencies were identified in ARV administration site for a child (P3: 23.1%) and laboratory referral for post-vaccination serology (P5: 26.0%). Intradermal ARV scheduling (P4: 83.7%), management of re-exposed vaccinated patients (P6: 88.5%), and RIG dilution technique (P9: 80.3%) were reasonably adequate.

### 3.6. Spearman Rank-Order Correlations—Overall Cohort

**Figure 4:**
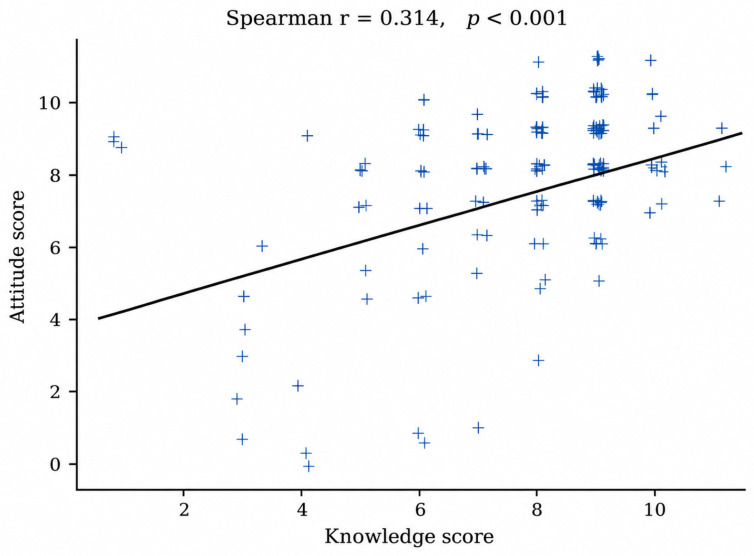
Spearman rank correlation—Knowledge vs. Attitude (r = 0.314, *p* < 0.001). Each "+" represents an individual respondent; solid line indicates the linear trend.

**Figure 5:**
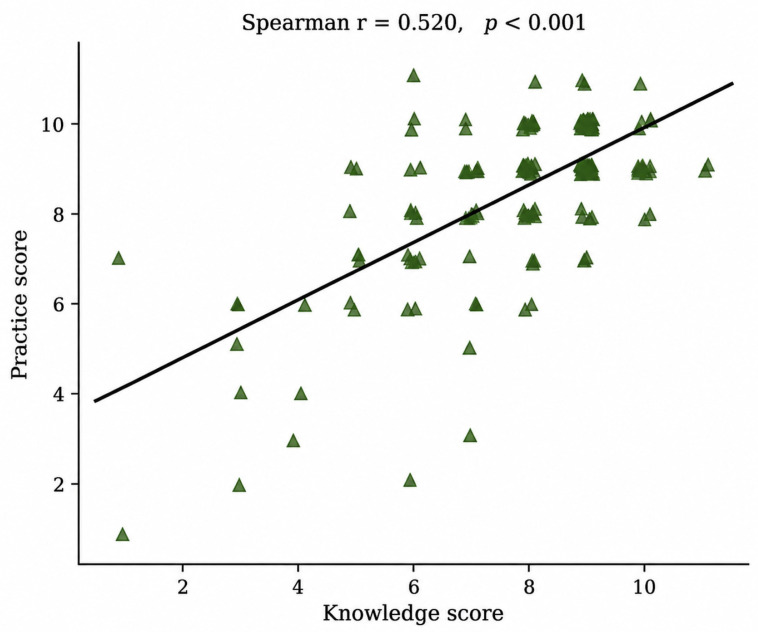
Spearman rank correlation—Knowledge vs. Practice (r = 0.520, *p* < 0.001). Each triangle represents an individual respondent; solid line indicates the linear trend.

**Figure 6:**
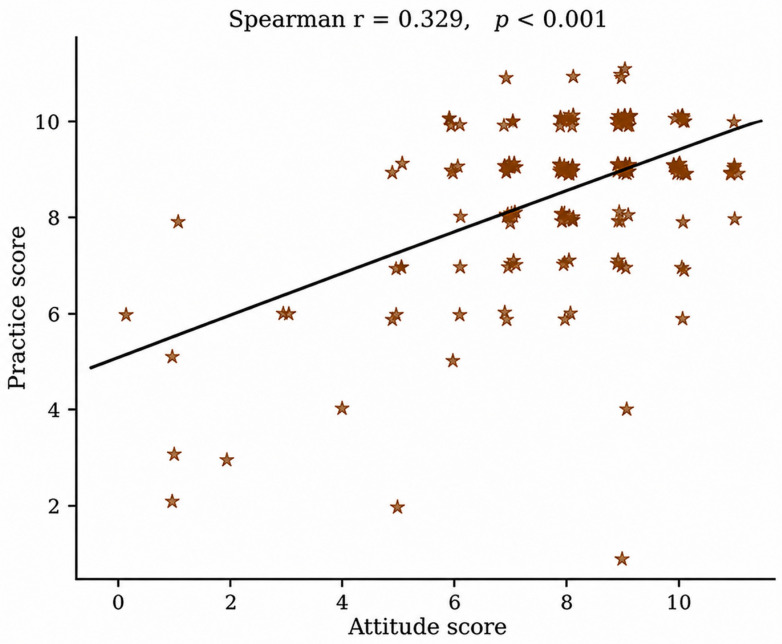
Spearman rank correlation—Attitude vs. Practice (r = 0.329, *p* < 0.001). Each star represents an individual respondent; solid line indicates the linear trend.

All three inter-domain correlations were positive and statistically significant (Table 6, Figure 4, Figure 5 and Figure 6). The strongest association was Knowledge–Practice (r = 0.520, *p* < 0.001), indicating that physicians with greater factual knowledge made more guideline-concordant clinical decisions. Moderate correlations were observed for Knowledge–Attitude (r = 0.314, *p* < 0.001) and Attitude–Practice (r = 0.329, *p* < 0.001), consistent with attitudinal beliefs partially mediating the knowledge-to-practice pathway.

### 3.7. KAP Scores Stratified by Specialty

**Figure 7:**
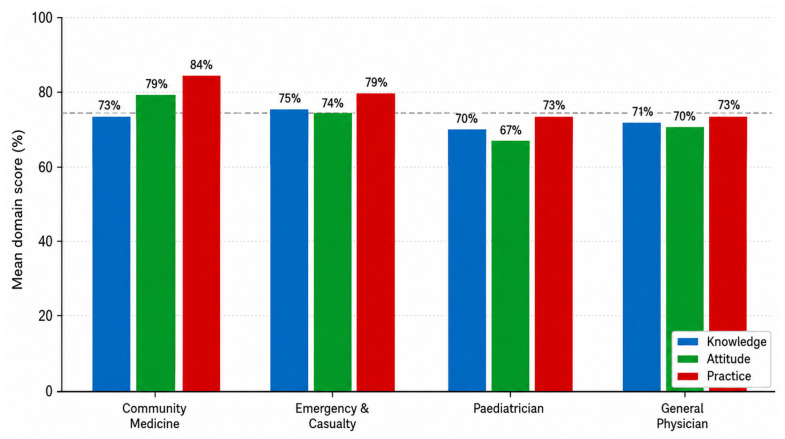
KAP Domain Scores (%) by Speciality. Dashed line = 75% benchmark.

Kruskal–Wallis testing revealed significant inter-specialty differences in Attitude (H = 16.15, *p* = 0.001) and Practice (H = 28.85, *p* < 0.001), but not Knowledge (H = 6.25, *p* = 0.100). Results are presented in Table 7 and Figure 7. Community Medicine specialists consistently excelled in all three domains (K 73.3%, A 79.1%, P 91.2%), likely due to their extensive public health training and familiarity with NCDC rabies guidelines. Emergency and Casualty physicians ranked second in Knowledge (75.1%) and Practice (85.4%), reflecting their frontline role in bite injury management. Pediatricians had the lowest Attitude score (66.7%), which is concerning since children bear a disproportionate burden of animal bite exposures in India. General Physicians exhibited the most variability among individuals, with an SD of up to 3.15 for Attitude, indicating a wide range of training backgrounds.

### 3.8. Stratified Spearman Correlations by Speciality

Spearman correlations stratified by speciality are presented in Table 8. The Knowledge–Practice correlation was significant and positive across all four speciality groups, with the strongest association observed among Emergency and Casualty Physicians (r = 0.595, *p* < 0.001), followed by General Physicians (r = 0.553, *p* = 0.040) and Pediatricians (r = 0.516, *p* < 0.001). Notably, the Attitude domain showed a weaker relationship to both Knowledge and Practice in Community Medicine practitioners (K-A r = 0.179, *p* = 0.134; A-P r = 0.065, *p* = 0.592), suggesting that their clinical practice may be more protocol-driven and less influenced by attitudinal variation. The 95% confidence intervals reported alongside each coefficient in Table 8 are wide for the General Physician subgroup (*n* = 14) and overlap zero for several coefficients across specialties, reflecting small subgroup sizes.

### 3.9. KAP Scores Stratified by State

**Figure 8:**
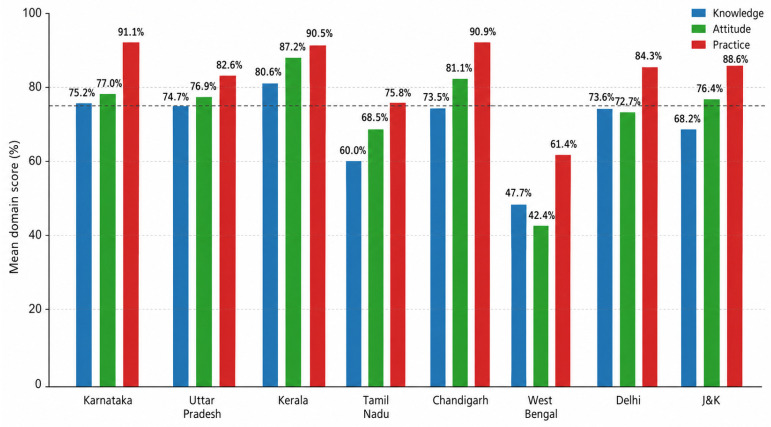
KAP Domain Scores (%) by State (*n* ≥ 10). Dashed line = 75% benchmark.

State-level results for states with *n* ≥ 5 are shown in Table 9 and Figure 8. Kruskal–Wallis tests revealed significant inter-state differences across all three domains (Knowledge: H = 49.51, *p* < 0.001; Attitude: H = 75.43, *p* < 0.001; Practice: H = 68.58, *p* < 0.001). Post hoc pairwise comparisons (Mann–Whitney U with Bonferroni correction) identified the following key findings. Kerala emerged as the best-performing state, with the highest Knowledge (80.6%) and Attitude (87.2%) scores and uniformly low intra-state variability. Karnataka and Chandigarh also performed strongly, particularly in Practice (91.1% and 90.9%, respectively), with Chandigarh showing remarkable uniformity (Practice SD = 0.00, all 12 respondents scoring 10/11). West Bengal was the lowest-performing state across all three domains (K 47.7%, A 42.4%, P 61.4%) and differed significantly from Karnataka, Uttar Pradesh, Kerala, Chandigarh, Jammu & Kashmir, and Assam in pairwise comparisons (all *p* < 0.05 after Bonferroni correction). Assam showed an interesting dissociation of excellent Knowledge (81.8%) and Practice (91.9%) alongside a markedly lower Attitude score (60.6%), suggesting that guideline-concordant clinical behaviour is not always mediated by correct attitudinal beliefs. Punjab showed a similar pattern with high Knowledge (76.6%) but low Attitude (50.6%).

### 3.10. Stratified Spearman Correlations by State (*n* ≥ 10)

Spearman correlations stratified by state (for states with *n* ≥ 10) are presented in Table 10. Uttar Pradesh demonstrated the most consistent and significant KAP inter-domain correlations (K-A: r = 0.580; K-P: r = 0.660; A-P: r = 0.669; all *p* < 0.001), indicating that knowledge, attitude, and practice are tightly coupled in this cohort. Kerala showed a significant K-A correlation (r = 0.502, *p* = 0.017) but non-significant K-P and A-P associations, which may reflect attitudinal homogeneity within this high-performing group. Chandigarh’s zero-value correlations likely reflect reduced variability due to a narrow scoring range in this small subgroup (*n* = 12). Delhi exhibited a notably high K-P correlation (r = 0.809, *p* = 0.003) despite its small sample size (*n* = 11). The 95% confidence intervals reported alongside each coefficient in Table 10 are wide, and in several cases cross zero, for states with *n* < 15 (Tamil Nadu, Chandigarh, West Bengal, Delhi, Jammu & Kashmir), indicating substantial sampling uncertainty; these state-stratified correlations have been interpreted with caution rather than as precise estimates of the true domain associations in each subgroup.

### 3.11. Comparative Analysis: 2026 Study vs. 2013 Benchmark

Item-level comparison between the 2013 benchmark study and the present cohort (*n* = 208, ten states) was performed using two-proportion Z-tests for matched items across all three domains.

Individual-level socio-demographic data (e.g., age, sex, years of clinical experience) for the 2013 benchmark cohort are not available in the published source, which precludes formal statistical balancing of socio-demographic factors, including physicians’ seniority, between the 2013 benchmark cohort and the 2026 survey cohort. However, both cohorts were restricted to physicians actively managing animal-bite exposures at anti-rabies clinics and included overlapping specialities (community medicine, emergency medicine, and general medicine), which supports broad comparability of the physician populations even though a formal covariate-adjusted comparison was not feasible. This constraint is discussed further as a source of potential selection bias in the Methods and Discussion sections. To aid this assessment, the specialty composition of the 2026 respondents (community medicine, emergency medicine, pediatrics, general physicians, other) is presented alongside the corresponding 2013 figures where available; where the 2013 source did not report a specialty-level breakdown, this is stated explicitly rather than shown as an incomplete numerical comparison.

### 3.12. Knowledge Domain Comparison

Five of eight matched Knowledge items demonstrated statistically significant improvements. Bite categorization increased by +38.3 percentage points (*p* < 0.001) and recognition of rabies-transmitting animals by +19.6 percentage points (*p* < 0.001). The most critical finding was a dramatic decline in knowledge of the IM PrEP schedule (Δ = −54.9 percentage points, *p* < 0.001), which may reflect guideline updates not adequately disseminated through curricula. ARV safety in pregnancy and dosing in infants showed minimal, non-significant changes, indicating stable performance at a high baseline (Table 11).

### 3.13. Attitude Domain Comparison

Six of eight matched Attitude items showed statistically significant differences. Large and highly significant decreases were observed in the categorization of animal contact as Category II exposure (Δ = −45.2, *p* < 0.001) and in the 10-day observation rule beyond dogs and cats (Δ = −43.8, *p* < 0.001), indicating major adverse shifts in attitude with direct clinical implications. Conversely, views on RIG/RmAb use (Δ = +22.3, *p* < 0.001) and intradermal vaccination equivalence (Δ = +15.9, *p* = 0.001) improved notably (Table 12).

### 3.14. Practice Domain Comparison

Six of seven matched Practice items showed significant improvement. The most notable gains were in PEP after delayed reporting (Δ = +29.7, *p* < 0.001), RIG site of administration (Δ = +21.8), RIG dilution (Δ = +22.6), and PEP for non-canine bites (Δ = +22.8; all *p* < 0.001). Only the management of Category III bites with uncontrolled bleeding did not reach significance (Δ = +2.4, *p* = 0.489). The Practice domain demonstrates the most consistent and substantial improvement over the 13-year period (Table 13).

### 3.15. Spearman Correlation Comparison

The Knowledge–Attitude correlation declined significantly (r: 0.667 to 0.314; Δr = −0.353, *p* = 0.0001), indicating a meaningful weakening of the association between physicians’ knowledge and their attitudes. The Knowledge–Practice correlation strengthened significantly (r: 0.220 to 0.520; Δr = +0.300, *p* = 0.003), reflecting a more direct translation of knowledge into clinical decision-making in the 2026 cohort. The Attitude–Practice correlation remained stable (r: 0.334 vs. 0.329; Δr = −0.005, *p* = 0.963) (Table 14).

## 4. Discussion

This multi-state, cross-sectional retrospective KAP survey of 208 Indian physicians provides a comprehensive assessment of current rabies PEP competency across various clinical specialities and ten states. This study constitutes the first national-level evaluation to include awareness of rabies monoclonal antibodies and a systematic comparison with the 2013 benchmark study.

The Practice domain achieved the highest mean score (78.5%), suggesting that scenario-based clinical decision-making is better retained than attitudinal alignment. Notably high scores for monkey-bite PEP (P1: 97.1%), delayed presentations (P8: 96.6%), and RIG/RmAb administration site (P7: 95.2%) indicate strong guideline adherence in common scenarios. However, two practice deficiencies demand immediate attention. First, only 23.1% correctly identified the ARV site for a 1.5-year-old child (P3); per WHO and NCDC 2019 guidelines, the anterolateral thigh is the correct injection site for children under two years [4,10]. Children bear a disproportionate burden of animal-bite exposures in India [2,11], making this a critical gap. Second, only 26.0% correctly identified NIMHANS Bangalore as the designated laboratory for post-vaccination serology, indicating systemic unfamiliarity with national rabies reference facilities.

The Knowledge domain mean (72.7%) reflected generally adequate understanding of core PEP principles, with strong performance on exposure categorization (K2: 94.2%), vaccine safety in pregnancy (K7: 85.6%), and single-agent RmAb dosing (K9: 82.2%). Two critical knowledge gaps were identified. K6—the IM PrEP schedule—was correctly answered by only 13.9% of respondents; a dramatic decline of 54.9% compared to the 2013 benchmark (68.8%). Pre-exposure prophylaxis is recommended for high-risk occupational groups including veterinary workers and laboratory personnel, and should be considered for children residing in remote, rabies-endemic regions where access to post-exposure prophylaxis is limited. K11—management of excess RIG was correctly answered by only 19.7%; WHO guidelines explicitly require that excess RIG be injected intramuscularly at a distant site if it cannot be fully infiltrated around the wound [4,10].

The Attitude domain mean (65.6%) reflected two large and clinically important deficiencies. Only 22.6% correctly identified touching or feeding animals as a Category I exposure, representing a 45.2 percentage point decline from 2013 (67.8%, *p* < 0.001). Only 34.1% accepted that the 10-day animal observation rule applies specifically to dogs and cats, declining to 43.8 points from 2013 (77.9%, *p* < 0.001). Both are explicitly specified in WHO and NCDC guidelines [4,10] and these attitudinal gaps, if translated into clinical practice, would lead to systematic under-treatment of animal exposures. Conversely, the Attitude domain showed significant improvements over 2013 in wound washing recognition (A6: +13.1 points), RIG/RmAb life-saving role (A9: +22.3 points, *p* < 0.001), and intradermal vaccination equivalence (A7: +15.9 points, *p* = 0.001).

The moderately strong Knowledge–Practice correlation (r = 0.520, *p* < 0.001) confirms that factual knowledge is a meaningful predictor of clinical decision quality and is a significant strengthening from the 2013 value of r = 0.220 (Δr = +0.300, *p* = 0.003), suggesting a more direct translation of knowledge into clinical decision-making in the current cohort. The Knowledge–Attitude correlation declined significantly (r: 0.667 in 2013 to 0.314 in 2026; *p* = 0.0001), implying that some physicians now hold attitudinal beliefs not fully concordant with their factual knowledge—a pattern consistent with the theory of planned behaviour [12,13,14]. The Attitude–Practice correlation remained stable (r = 0.329 vs. 0.334; *p* = 0.963).

Speciality significantly predicted Attitude (H = 16.15, *p* = 0.001) and Practice performance (H = 28.85, *p* < 0.001). The best performance across all domains was achieved by community medicine specialists (Practice 91.2%), as expected, given their public health training and familiarity with NCDC guidelines. The Emergency and Casualty physicians (r = 0.595, *p* < 0.001) exhibited strong knowledge–practice correlation reflecting their frontline role. Pediatricians showed the lowest Practice score (79.0%), and the Attitude domain showed a weaker relationship to practice in Community Medicine (A-P r = 0.065, *p* = 0.592), suggesting protocol-driven rather than attitude-mediated behaviour. The widest inter-individual variability (Attitude SD = 3.15) was observed in general physicians.

State-level analysis revealed significant variation across all three domains. Kerala demonstrated consistently high scores (K 80.6%, A 87.2%, P 90.5%), consistent with its well-established public health infrastructure. Uttar Pradesh demonstrated the most consistent KAP inter-domain correlations (all r > 0.58, *p* < 0.001), indicating tightly coupled knowledge, attitudes, and practice in this cohort. West Bengal scored lowest across all three domains (K 47.7%, A 42.4%, P 61.4%) and was significantly different from all other major states. Assam showed an interesting dissociation of excellent Knowledge (81.8%) and Practice (91.9%) alongside a markedly lower Attitude score (60.6%), a pattern also observed in Punjab (K 76.6%, A 50.6%), suggesting that guideline-concordant behaviour in these cohorts is protocol-driven rather than attitude-mediated.

Comparing these findings with KAP studies conducted among physicians in other settings provides useful context for the observed scores. Within India, an earlier physician-focused survey in Delhi reported considerably lower awareness of the intradermal PEP schedule, site, and dose than the Practice-domain performance seen in the present cohort, though that comparator was restricted to a single city and predates the present study by over a decade [15]. Internationally, a 2024 survey of healthcare professionals in Peshawar, Pakistan, similarly found strong general awareness of rabies transmission and symptoms alongside a specific, persistent gap in recognizing less common transmission routes, a pattern broadly comparable to the good foundational knowledge but persistent guideline-level gaps (e.g., IM PrEP scheduling, RIG management) identified here [16]. A cross-sectional survey of human and animal health professionals in Senegal found that fewer than half demonstrated sufficient knowledge, positive attitudes, or good practice regarding rabies overall, considerably lower than the domain scores reported in the present cohort [17]. Plausible explanations for India’s comparatively stronger Practice-domain performance include a longer-established national PEP infrastructure of dedicated anti-rabies clinics, NCDC-issued guidelines, and continuing medical education coordinated through APCRI, together with the present study’s focus on physicians already actively managing bite exposures rather than a general healthcare workforce with less routine PEP exposure. Conversely, the comparatively modest Attitude-domain performance in this cohort, particularly the low endorsement of Category II animal-contact exposure and the dogs/cats-only observation rule, suggests that attitudinal training may be less consistently reinforced than procedural/practice training in existing Indian CME curricula. Direct cross-country comparison remains limited by differences in questionnaire structure, scoring thresholds, and study populations and should be interpreted with this caveat in mind.

Acceptance of RmAbs as safe (A11: 84.6%) and effective alternatives to RIGs (A10: 88.5%) among surveyed physicians is a notable finding. These perceptions are broadly consistent with properties reported in the published literature, including standardized dosing, absence of serum-sickness risk, and manufacturing scalability [18,19,20,21]. These data suggest that attitudinal barriers to RmAb adoption are low and that primary constraints are likely operational. Knowledge of single-agent RmAb dose was strong (K9: 82.2%), while cocktail-preparation dosing was lower (K10: 68.8%), representing a trainable knowledge gap.

Rabies control strategies in India have evolved over the past two decades, with the Association for Prevention and Control of Rabies in India (APCRI) contributing to national multicentric surveys, updated clinical guidelines, and continuing medical education for physicians managing animal-bite exposures. Post-marketing surveillance studies conducted in India have reported low rates of adverse events with RmAb use: a pan-India surveillance of 1000 patients receiving Rabishield with Rabivax-S reported adverse events in 6.4% of patients, with one unrelated serious adverse event and no cases of breakthrough rabies [20], and separate post-marketing studies of TwinRab in cohorts of 401 and 215 patients reported only mild, self-limiting local reactions and no treatment-related serious adverse events [21]. We note that these post-marketing studies were conducted or supported by the manufacturer of the study vaccine, consistent with the declared conflicts of interest for this manuscript, and this safety literature should be interpreted with that affiliation in mind. Given the cross-sectional design of the present survey, the high RmAb acceptance and the strengthened Knowledge–Practice correlation observed in this cohort relative to the 2013 benchmark cannot be directly attributed to any specific educational or industry initiative; the association reported here is descriptive rather than causal. This study has several strengths, such as the largest multi-state Indian KAP survey on rabies PEP to date; includes four major specialties across ten states; is the first national assessment of RmAb awareness; and provides a systematic 13-year temporal comparison using matched items. Limitations include potential selection bias from voluntary electronic participation via a professional conference platform rather than a defined per-state sampling frame, which likely resulted in disproportionate representation of states with larger anti-rabies clinic networks or higher conference attendance, non-response bias, inability to verify self-reported practice against clinical records, and limited sample sizes for individual states with *n* < 10. Correlation analyses stratified by specialty and state (Table 8 and Table 10) are similarly limited by small subgroup sizes in some strata (e.g., General Physicians, *n* = 14; several states with *n* < 15), which widen the corresponding 95% confidence intervals and warrant cautious interpretation of those subgroup-level associations. Differences in specialty mix between the 2013 and 2026 cohorts (Table 11, Table 12, Table 13 and Table 14) may also partly confound the temporal comparison. The study did not collect additional socio-demographic variables (e.g., age, sex, years of clinical experience) beyond specialty and state, which limits our ability to assess balance on those dimensions between the two cohorts.

## 5. Conclusions

This multi-state, cross-sectional retrospective KAP survey of Indian physicians found overall rabies PEP competency to be adequate, with Practice scoring highest (78.5%), Knowledge intermediate (72.7%), and Attitude lowest (65.6%). Relative to the 2013 benchmark, Practice showed the most consistent gains, while Knowledge and Attitude improved on some items but declined on others, notably the pre-exposure prophylaxis schedule and recognition of Category II exposures. Acceptance of rabies monoclonal antibodies was high, with most physicians recognizing them as safe, effective alternatives to conventional immunoglobulins, indicating that attitudinal barriers to their use are low and that the main constraints to wider RmAb administration are likely knowledge- and system-level rather than attitudinal. To further increase guideline-concordant RmAb use, continuing medical education should specifically target RmAb dosing, management of excess RIG/RmAb after wound infiltration, and accurate exposure categorization, delivered through APCRI-coordinated, specialty- and state-specific training prioritizing the groups identified here with the largest gaps.

## Figures and Tables

**Table 1:** Participant Distribution by Specialty and State.

Category	*n*	%
By Speciality		
Community Medicine	71	34.1
Emergency Medicine Physicians	58	27.9
Pediatricians	44	21.2
General Physicians	14	6.7
Others including Surgery	21	10.1
By State (*n* ≥ 10)		
Karnataka	45	21.6
Uttar Pradesh	37	17.8
Kerala	22	10.6
Tamil Nadu	15	7.2
Chandigarh	12	5.8
West Bengal	12	5.8
Delhi	11	5.3
Jammu & Kashmir	10	4.8
Other states (*n* < 10 each)	44	21.1

**Table 2:** KAP Domain-Level Descriptive Statistics (*n* = 208).

Domain	Max Score	Mean ± SD	Mean %	W	*p* (Normality)
Knowledge	11	8.00 ± 1.69	72.7%	0.835	<0.0001
Attitude	11	7.22 ± 1.74	65.6%	0.863	<0.0001
Practice	11	8.64 ± 1.65	78.5%	0.795	<0.0001

SD: Standard Deviation.

**Table 3:** Knowledge Domain: Item-Level Correct Response Rates (*n* = 208).

Item	Knowledge Item	Correct (*n*)	% Correct
K1	Animals that do not transmit rabies (domestic rodents)	178	86.5%
K2	Category of exposure for a single wound with bleeding (Cat III)	196	94.2%
K3	Dose of anti-rabies vaccine (ARV) in infants (same as adult dose)	180	86.5%
K4	Site of administration of ARV in adults (deltoid)	191	91.8%
K5	IM PEP schedule per NCDC guidelines (Days 0, 3, 7, 14 & 28)	181	87.0%
K6	IM PrEP schedule per NCDC guidelines	29	13.9%
K7	Safety of ARV in pregnancy and lactation (Yes)	178	85.6%
K8	Dose of Equine Rabies Immunoglobulin—ERIG (40 IU/Kg)	173	83.2%
K9	Dose of single rabies monoclonal antibody (3.33 IU/Kg)	171	82.2%
K10	Dose of cocktail of two rabies monoclonal antibodies (40 IU/Kg)	143	68.8%
K11	Management of excess RIG after wound infiltration (Category III)	41	19.7%

ARV: Anti Rabies Vaccine; ERIG: Equine Rabies Immunoglobulin; IM PEP: Intramuscular Post-Exposure Prophylaxis; IM PrEP: Intramuscular Pre-Exposure Prophylaxis; IU: International Unit; NCDC: National Centre for Disease Control; RIG: Rabies Immunoglobulin.

**Table 4:** Attitude Domain: Item-Level Correct Response Rates (*n* = 208).

Item	Attitude Statement	Correct (*n*)	% Correct
A1	Andaman & Nicobar Islands are free from rabies (Agree)	175	84.1%
A2	Rabies is transmitted by the bite of squirrels (Disagree)	123	59.1%
A3	Touching/feeding of animals is Category I exposure (Agree)	47	22.6%
A4	Pet dogs do not transmit rabies (Disagree)	179	86.1%
A5	Observation of animals applies only to dogs & cats (Agree)	71	34.1%
A6	Wound washing with soap & water reduces viral load (Agree)	195	93.8%
A7	Intradermal rabies vaccine is less effective than IM (Disagree)	174	83.7%
A8	ARV can also be given subcutaneously (Disagree)	153	73.6%
A9	RIGs & RmAbs are lifesaving in Category III bites (Agree)	199	95.7%
A10	RmAbs are alternative/effective replacements for RIGs (Agree)	184	88.5%
A11	Rabies Monoclonal Antibodies are safer than RIGs (Agree)	176	84.6%

ARV: Anti Rabies Vaccine; IM: Intramuscular; IU: International Unit; RIG: Rabies Immunoglobulin; RmAbs: Rabies Monoclonal Antibodies.

**Table 5:** Practice Domain: Item-Level Correct Response Rates (*n* = 208).

Item	Practice Scenario	Correct (*n*)	% Correct
P1	PEP management for a monkey bite	202	97.1%
P2	Management of pregnant woman with Category II dog bite	194	93.3%
P3	ARV administration site in a child (anterolateral thigh)	48	23.1%
P4	IDRV schedule per NCDC guidelines (Days 0, 3, 7 & 28)	174	83.7%
P5	Laboratory for serum antibody response to vaccine (NIMHANS, Bangalore)	54	26.0%
P6	PEP for a previously vaccinated person bitten again	184	88.5%
P7	Correct site for RIG/RmAb administration	198	95.2%
P8	Management when patient presents 6 days post-exposure	201	96.6%
P9	Management when RIG volume is insufficient (dilute with NS)	167	80.3%
P10	Management of Category III bite with uncontrolled bleeding	190	91.3%
P11	Category III bite by vaccinated pet dog—recommended prophylaxis	185	88.9%

ARV: Anti Rabies Vaccine; ID: Intradermal; IDRV: Intradermal Rabies Vaccination; NCDC: National Centre for Disease Control; RIG: Rabies Immunoglobulin; RmAbs: Rabies Monoclonal Antibodies; PEP: Post-Exposure Prophylaxis.

**Table 6:** Spearman Rank-Order Correlation Matrix—Overall Cohort (*n*= 208).

Domain Pair	r	*p*-Value	Interpretation
Knowledge–Attitude	0.314; [95% CI: 0.186, 0.432]	<0.001	Moderate, significant
Knowledge–Practice	0.520; [95% CI: 0.413, 0.613]	<0.001	Moderately strong, significant
Attitude–Practice	0.329; [95% CI: 0.202, 0.445]	<0.001	Moderate, significant

**Table 7:** KAP Domain Scores Stratified by Specialty.

Specialty	*n*	Knowledge Mean ± SD (%)	Attitude Mean ± SD (%)	Practice Mean ± SD (%)
Community Medicine	71	8.06 ± 1.47 (73.3%)	8.70 ± 1.09 (79.1%)	10.03 ± 0.89 (91.2%)
Emergency and Casualty	58	8.26 ± 1.74 (75.1%)	8.10 ± 2.17 (73.7%)	9.40 ± 1.47 (85.4%)
Pediatrician	44	7.76 ± 1.63 (70.5%)	7.33 ± 1.99 (66.7%)	8.69 ± 1.89 (79.0%)
General Physician	14	7.86 ± 2.60 (71.5%)	7.64 ± 3.15 (69.5%)	8.36 ± 2.76 (76.0%)

SD: Standard Deviation.

**Table 8:** Spearman Rank Correlations Stratified by Specialty.

Specialty	*n*	K–A (r; *p*)	K–P (r; *p*)	A–P (r; *p*)
Community Medicine	71	0.179; *p* = 0.134; 95% CI [−0.057, 0.396]	0.362; *p* = 0.002; 95% CI [0.141, 0.549]	0.065; *p* = 0.592; 95% CI [−0.171, 0.294]
Emergency and Casualty	58	0.437; *p* = 0.001; 95% CI [0.201, 0.625]	0.595; *p* < 0.001; 95% CI [0.398, 0.740]	0.304; *p* = 0.020; 95% CI [0.050, 0.521]
Pediatrician	44	0.200; *p* = 0.194; 95% CI [−0.103, 0.469]	0.516; *p* < 0.001; 95% CI [0.259, 0.705]	0.459; *p* = 0.002; 95% CI [0.188, 0.665]
General Physician	14	0.523; *p* = 0.055; 95% CI [−0.011, 0.825]	0.553; *p* = 0.040; 95% CI [0.032, 0.838]	0.497; *p* = 0.071; 95% CI [−0.046, 0.813]

A: Attitude; K: Knowledge; P: Practice.

**Table 9:** KAP Domain Scores by State (Mean ± SD; *n* = 208; *n* ≥ 5 shown).

State	*n*	Knowledge Mean ± SD	K %	Attitude Mean ± SD	A %	Practice Mean ± SD	P %
Karnataka	45	8.27 ± 1.05	75.2%	8.47 ± 1.69	77.0%	10.02 ± 1.50	91.1%
Uttar Pradesh	37	8.22 ± 1.72	74.7%	8.46 ± 1.30	76.9%	9.08 ± 1.59	82.6%
Kerala	22	8.86 ± 0.64	80.6%	9.59 ± 1.53	87.2%	9.95 ± 0.38	90.5%
Tamil Nadu	15	6.60 ± 2.77	60.0%	7.53 ± 1.19	68.5%	8.33 ± 2.32	75.8%
Chandigarh	12	8.08 ± 0.67	73.5%	8.92 ± 0.29	81.1%	10.00 ± 0.00	90.9%
West Bengal	12	5.25 ± 2.26	47.7%	4.67 ± 2.74	42.4%	6.75 ± 1.96	61.4%
Delhi	11	8.09 ± 1.04	73.6%	8.00 ± 1.18	72.7%	9.27 ± 0.90	84.3%
Jammu & Kashmir	10	7.50 ± 1.43	68.2%	8.40 ± 0.84	76.4%	9.70 ± 1.42	88.2%
Assam	9	9.00 ± 0.00	81.8%	6.67 ± 0.71	60.6%	10.11 ± 0.60	91.9%
Punjab	7	8.43 ± 1.62	76.6%	5.57 ± 3.78	50.6%	8.14 ± 2.34	74.0%
Odisha	6	8.00 ± 1.41	72.7%	8.83 ± 1.17	80.3%	9.83 ± 0.98	89.4%
Maharashtra	5	8.00 ± 2.00	72.7%	7.80 ± 0.84	70.9%	9.20 ± 1.64	83.6%
Gujarat	4	7.50 ± 1.73	68.2%	7.00 ± 1.41	63.6%	8.75 ± 1.26	79.5%
Rajasthan	4	8.00 ± 0.82	72.7%	8.75 ± 0.50	79.5%	9.75 ± 0.50	88.6%
Telangana	2	9.00 ± 0.00	81.8%	8.00 ± 1.41	72.7%	9.50 ± 2.12	86.4%
Haryana	2	9.50 ± 0.71	86.4%	8.50 ± 0.71	77.3%	8.50 ± 0.71	77.3%
* Other states	5	8.40 ± 1.95	76.4%	7.20 ± 1.10	65.5%	10.20 ± 0.45	92.7%

* Other states (*n* = 1 each): Andhra Pradesh, Bihar, Chhattisgarh, Manipur, Nagaland. A: Attitude; K: Knowledge; P: Practice; SD: Standard Deviation.

**Table 10:** Spearman Correlations Stratified by State (*n* ≥ 10).

State	*n*	K–A (r; *p*)	K–P (r; *p*)	A–P (r; *p*)
Karnataka	45	0.363; *p* = 0.014; 95% CI [0.078, 0.593]	0.251; *p* = 0.096; 95% CI [−0.046, 0.507]	0.204; *p* = 0.180; 95% CI [−0.095, 0.469]
Uttar Pradesh	37	0.580; *p* < 0.001; 95% CI [0.315, 0.761]	0.660; *p* < 0.001; 95% CI [0.427, 0.811]	0.669; *p* < 0.001; 95% CI [0.440, 0.816]
Kerala	22	0.502; *p* = 0.017; 95% CI [0.102, 0.762]	0.061; *p* = 0.788; 95% CI [−0.370, 0.471]	−0.051; *p* = 0.823; 95% CI [−0.463, 0.379]
Tamil Nadu	15	−0.253; *p* = 0.363; 95% CI [−0.677, 0.298]	0.527; *p* = 0.043; 95% CI [0.020, 0.818]	−0.336; *p* = 0.221; 95% CI [−0.724, 0.213]
Chandigarh	12	0.000; *p* = 1.000; 95% CI [−0.574, 0.574]	0.000; *p* = 1.000; 95% CI [−0.574, 0.574]	0.174; *p* = 0.588; 95% CI [−0.444, 0.680]
West Bengal	12	0.601; *p* = 0.039; 95% CI [0.041, 0.874]	0.456; *p* = 0.136; 95% CI [−0.160, 0.816]	0.383; *p* = 0.219; 95% CI [−0.245, 0.784]
Delhi	11	0.054; *p* = 0.875; 95% CI [−0.564, 0.633]	0.809; *p* = 0.003; 95% CI [0.406, 0.949]	0.241; *p* = 0.476; 95% CI [−0.420, 0.735]
Jammu & Kashmir	10	0.042; *p* = 0.908; 95% CI [−0.604, 0.654]	0.606; *p* = 0.063; 95% CI [−0.038, 0.894]	0.341; *p* = 0.336; 95% CI [−0.368, 0.799]

A: Attitude; K: Knowledge; P: Practice.

**Table 11:** Knowledge Domain Comparison: 2013 (*n* = 109) vs. 2026 (*n* = 208).

Item	Question	2013%	2026%	Δ	Z	*p*-Value
K1	Animals that do not transmit rabies	66.9	86.5	+19.6	−4.13	<0.001
K2	Category of exposure for a single wound with bleeding	55.9	94.2	+38.3	−8.26	<0.001
K3	Dose of anti-rabies vaccine (ARV) in infants	84.4	86.5	+2.1	−0.51	0.611
K4	Site of administration of ARV in adults	83.5	91.8	+8.3	−2.24	0.025
K5	IM PEP schedule per NCDC guidelines	74.4	87.0	+12.6	−2.82	0.005
K6	IM PrEP schedule per NCDC guidelines	68.8	13.9	−54.9	+9.89	<0.001
K7	Safety of ARV in pregnancy and lactation	85.3	85.6	+0.3	−0.07	0.943
K8	Dose of Equine Rabies Immunoglobulin (ERIG)	66.9	83.2	+16.3	−3.31	<0.001

ARV: Anti Rabies Vaccine; ERIG: Equine Rabies Immunoglobulin; IM PEP: Intramuscular Post-Exposure Prophylaxis; IM PrEP: Intramuscular Pre-Exposure Prophylaxis; NCDC: National Centre for Disease Control.

**Table 12:** Attitude Domain Comparison: 2013 (*n* = 109) vs. 2026 (*n* = 208).

Item	Statement (Correct Response)	2013%	2026%	Δ	Z	*p*-Value
A2	Rabies transmitted by squirrel bites (Disagree)	75.3	59.1	−16.2	+2.87	0.004
A3	Touching/feeding animals is Category I (Agree)	67.8	22.6	−45.2	+7.87	<0.001
A4	Pet dogs do not transmit rabies (Disagree)	92.7	86.1	−6.6	+1.74	0.082
A5	Observation applies only to dogs and cats (Agree)	77.9	34.1	−43.8	+7.41	<0.001
A6	Wound washing reduces virus load (Agree)	80.7	93.8	+13.1	−3.58	<0.001
A7	ID vaccine less effective than IM (Disagree)	67.8	83.7	+15.9	−3.26	0.001
A8	ARV given subcutaneously (Disagree)	78.9	73.6	−5.3	+1.04	0.298
A9	RIGs and RmAbs lifesaving in Cat III (Agree)	73.4	95.7	+22.3	−5.81	<0.001

ARV: Anti Rabies Vaccine; ID: Intradermal; IM: Intramuscular; NCDC: National Centre for Disease Control; RIG: Rabies Immunoglobulin; RmAbs: Rabies Monoclonal Antibodies.

**Table 13:** Practice Domain Comparison: 2013 (*n* = 109) vs. 2026 (*n* = 208).

Item	Scenario	2013%	2026%	Δ	Z	*p*-Value
P1	PEP for a monkey bite	74.3	97.1	+22.8	−6.23	<0.001
P4	ID ARV schedule	68.9	83.7	+14.8	−3.05	0.002
P6	PEP for previously vaccinated re-exposed person	68.9	88.5	+19.6	−4.29	<0.001
P7	Correct site for RIG/RmAb administration	73.4	95.2	+21.8	−5.61	<0.001
P8	Management when patient presents 6 days post-exposure	66.9	96.6	+29.7	−7.33	<0.001
P9	Management when RIG volume is insufficient	57.7	80.3	+22.6	−4.28	<0.001
P10	Category III bite with uncontrolled bleeding	88.9	91.3	+2.4	−0.69	0.489

ARV: Anti Rabies Vaccine; ID: Intradermal; PEP: Post-Exposure Prophylaxis RIG: Rabies Immunoglobulin; RmAbs: Rabies Monoclonal Antibodies.

**Table 14:** Spearman Correlation Comparison: 2013 vs. 2026.

Domain Pair	r (2013)	r (2026)	Δr	Z-Diff	*p*-Value
Knowledge–Attitude	0.667	0.314	−0.353	4.02	0.0001
Knowledge–Practice	0.220	0.520	+0.300	−2.95	0.003
Attitude–Practice	0.334	0.329	−0.005	0.05	0.963

## Data Availability

The datasets used and/or analyzed during the current study will be available from the corresponding author on reasonable request.

## Abbreviations

The following abbreviations are used in this manuscript:

APCRI	Association for Prevention and Control of Rabies in India
ARV	Anti-Rabies Vaccine
CME	Continuing Medical Education
DHSPL	Digicare Health Solutions Private Limited
ERIG	Equine Rabies Immunoglobulin
GCP	Good Clinical Practice
ICMR	Indian Council of Medical Research
ID	Intradermal
IDRV	Intradermal Rabies Vaccination
IM	Intramuscular
IU	International Unit
KAP	Knowledge, Attitude and Practice
NCDC	National Centre for Disease Control
NIMHANS	National Institute of Mental Health and Neurosciences
NS	Normal Saline
PEP	Post-Exposure Prophylaxis
PMS	Post-Marketing Surveillance
PrEP	Pre-Exposure Prophylaxis
RIG	Rabies Immunoglobulin
RmAbs	Rabies Monoclonal Antibodies
SD	Standard Deviation
SPSS	Statistical Package for the Social Sciences
WHO	World Health Organization

## References

[1] Liu X., Zha Y., Wang Z., Jiang Y., Zhang X., Guo J., Li J., Zhang Q., Tsao E. (2025). The Pharmacokinetics and Safety Comparison of Zamerovimab and Mazorelvimab Monoclonal Antibodies vs. HRIG in Category III Rabies Post-Exposure Prophylaxis: A Stratified Analysis by Wound Characteristics. Biologicals.

[2] Sudarshan M.K., Madhusudana S.N., Mahendra B.J., Rao N.S.N., Ashwath Narayana D.H., Abdul Rahman S., Meslin F.-X., Lobo D., Ravikumar K. (2007). Gangaboraiah Assessing the Burden of Human Rabies in India: Results of a National Multi-Center Epidemiological Survey. Int. J. Infect. Dis..

[3] Ichhpujani R.L., Mala C., Veena M., Singh J., Bhardwaj M., Bhattacharya D., Pattanaik S.K., Balakrishnan N., Reddy A.K., Sampath G. (2008). Epidemiology of Animal Bites and Rabies Cases in India: A Multicentric Study. J. Commun. Dis..

[4] World Health Organization (2018). WHO Expert Consultation on Rabies: Third Report.

[5] Shankaraiah R.H., Bilagumba G., Narayana D., Annadani R., Vijayashankar V. (2013). Knowledge, Attitude, and Practice of Rabies Prophylaxis among Physicians at Indian Animal Bite Clinics. Asian Biomed..

[6] Sparrow E., Torvaldsen S., Newall A.T., Wood J.G., Sheikh M., Kieny M.P., Abela-Ridder B. (2019). Recent Advances in the Development of Monoclonal Antibodies for Rabies Post-Exposure Prophylaxis: A Review of the Current Status of the Clinical Development Pipeline. Vaccine.

[7] Pardeshi G., Sharma P., Ittiel A. (2026). Rabies Post-Exposure Prophylaxis in India: A SWOT Analysis. Ther. Adv. Vaccines Immunother..

[8] Kaliyaperumal K. (2004). Guideline for Conducting a Knowledge, Attitude and Practice (KAP) Study. AECS Illum..

[9] Gibson A.D., Yale G., Corfmat J., Appupillai M., Gigante C.M., Lopes M., Betodkar U., Costa N.C., Fernandes K.A., Mathapati P. (2022). Elimination of Human Rabies in Goa, India through an Integrated One Health Approach. Nat. Commun..

[10] Directorate General of Health Services, Ministry of Health & Family Welfare, Government of India (2019). National Guidelines for Rabies Prophylaxis.

[11] Sudarshan M.K., Mahendra B.J., Madhusudana S.N., Ashwoath Narayana D.H., Rahman A., Rao N.S.N., X-Meslin F., Lobo D., Ravikumar K. (2006). Gangaboraiah An Epidemiological Study of Animal Bites in India: Results of a WHO Sponsored National Multi-Centric Rabies Survey. J. Commun. Dis..

[12] Festinger L. (1957). A Theory of Cognitive Dissonance.

[13] Launiala A. (2009). How Much Can a KAP Survey Tell Us about People’s Knowledge, Attitudes and Practices? Some Observations from Medical Anthropology Research on Malaria in Pregnancy in Malawi. Anthr. Matters J..

[14] Ajzen I. (1991). The Theory of Planned Behavior. Organ. Behav. Hum. Decis. Process.

[15] Garg A., Kumar R., Ingle G.K. (2013). Knowledge and Practices Regarding Animal Bite Management and Rabies Prophylaxis among Doctors in Delhi, India. Asia Pac. J. Public Health.

[16] Ahmad A., Inayat F., Ullah N., Rasul S., Bakhtiar S., Shad Z., Ahmad Z. (2024). Knowledge, Attitudes, and Practices of Healthcare Professionals Regarding Rabies in Tertiary Care Hospitals: A Cross-Sectional Study in Peshawar, Pakistan. PLoS Negl. Trop. Dis..

[17] Ba M.F., Kane N.M., Diallo M.K.K., Bassoum O., Boh O.K., Mboup F.Z.M., Faye E.H.B., Bedekelabou A.P., Dieng S.D., Diop F.N. (2021). Knowledge, Attitudes and Practices on Rabies among Human and Animal Health Professionals in Senegal. Pathogens.

[18] Fan L., Zhang L., Li J., Zhu F. (2022). Advances in the Progress of Monoclonal Antibodies for Rabies. Hum. Vaccin. Immunother..

[19] Kim P.K., Ahn J.S., Kim C.M., Seo J.M., Keum S.J., Lee H.J., Choo M.J., Kim M.S., Lee J.Y., Maeng K.E. (2021). A Broad-Spectrum and Highly Potent Human Monoclonal Antibody Cocktail for Rabies Prophylaxis. PLoS ONE.

[20] Kang G., Lakhkar A., Bhamare C., Dharmadhikari A., Narwadkar J., Kanujia A., Kapse D., Gunale B., Poonawalla C.S., Kulkarni P.S. (2023). Active Safety Surveillance of Rabies Monoclonal Antibody and Rabies Vaccine in Patients with Category III Potential Rabies Exposure. Lancet Reg. Health Southeast Asia.

[21] Manna A., Kundu A.K., Sharma Sarkar B., Maji B., Dutta T., Mahajan M. (2024). Real-World Safety of TwinRab, the World’s First Novel Cocktail of Rabies Monoclonal Antibodies, in a Clinical Setting. Cureus.

